# Profiling parvalbumin interneurons using iPSC: challenges and perspectives for Autism Spectrum Disorder (ASD)

**DOI:** 10.1186/s13229-020-0314-0

**Published:** 2020-01-29

**Authors:** Federica Filice, Beat Schwaller, Tanja M. Michel, Edna Grünblatt

**Affiliations:** 1 Department of Neuroscience & Movements Science, Section of Medicine, University of Fribourg, Fribourg, Switzerland; 2 Department of Psychiatry, Department of Clinical Research, University of Southern Denmark, Odense, Denmark; 3 Psychiatry in the Region of Southern Denmark, Department of Psychiatry, Odense University Hospital Southern Denmark, Odense, Denmark; 4 Research Unit for Psychiatry Odense, Institute for Clinical Research, University of Southern Denmark, Odense, Denmark; 5 Department of Child and Adolescent Psychiatry and Psychotherapy, University Hospital of Psychiatry, University of Zurich, Neumuensterallee 3, 8032 Zurich, Switzerland; 6 Neuroscience Center Zurich, University of Zurich and ETH Zurich, Winterthurerstr. 190, 8057 Zurich, Switzerland; 7 Zurich Center for Integrative Human Physiology, University of Zurich, Winterthurerstr. 190, 8057 Zurich, Switzerland

**Keywords:** Parvalbumin, Induced pluripotent stem cells, Autism spectrum disorder, Schizophrenia, CRISPR-Cas9 technology, GABAergic, Interneuron

## Abstract

Autism spectrum disorders (ASD) are persistent conditions resulting from disrupted/altered neurodevelopment. ASD multifactorial etiology—and its numerous comorbid conditions—heightens the difficulty in identifying its underlying causes, thus obstructing the development of effective therapies. Increasing evidence from both animal and human studies suggests an altered functioning of the parvalbumin (PV)-expressing inhibitory interneurons as a common and possibly unifying pathway for some forms of ASD. PV-expressing interneurons (short: PVALB neurons) are critically implicated in the regulation of cortical networks’ activity. Their particular connectivity patterns, i.e., their preferential targeting of perisomatic regions and axon initial segments of pyramidal cells, as well as their reciprocal connections, enable PVALB neurons to exert a fine-tuned control of, e.g., spike timing, resulting in the generation and modulation of rhythms in the gamma range, which are important for sensory perception and attention.

New methodologies such as induced pluripotent stem cells (iPSC) and genome-editing techniques (CRISPR/Cas9) have proven to be valuable tools to get mechanistic insight in neurodevelopmental and/or neurodegenerative and neuropsychiatric diseases. Such technological advances have enabled the generation of PVALB neurons from iPSC. Tagging of these neurons would allow following their fate during the development, from precursor cells to differentiated (and functional) PVALB neurons. Also, it would enable a better understanding of PVALB neuron function, using either iPSC from healthy donors or ASD patients with known mutations in ASD risk genes. In this concept paper, the strategies hopefully leading to a better understanding of PVALB neuron function(s) are briefly discussed. We envision that such an iPSC-based approach combined with emerging (genetic) technologies may offer the opportunity to investigate in detail the role of PVALB neurons and PV during “neurodevelopment ex vivo.”

## Background

Autism spectrum disorder (ASD) is a pervasive neurodevelopmental disorder, characterized by impaired social interaction and communication, as well as restricted and/or repetitive behaviors and interests [1]. Despite many years of research, the multifactorial etiology of ASD hampers the elucidation of its underlying neurobiology, thus resulting in limited therapeutic approaches for ASD-diagnosed patients.

Hundreds of ASD-risk genes have been identified, many of which coding for synaptic-related proteins; however, rare variants with large effects account for less than 1% of cases of autism [2, 3], while a greater combination of common variants with small effects was found in the sporadic ASD patients [4]. In addition, environmental factors most likely causing epigenetic alterations were found to contribute to the etiology of ASD [5].

Interestingly, increasing evidence from both animal and human studies suggests an altered functioning of the parvalbumin (PV)-expressing subgroup of GABAergic interneurons as a common and possibly unifying pathway for some forms of ASD (see [6, 7]). PV interneurons (short: PVALB neurons) are key regulators of the activity of cortical networks, notably the oscillatory activity in the gamma-frequency range (30–80 Hz) [8–11]; therefore, the integrity of neuronal circuits containing these neurons is essential for the physiological functioning of the entire brain.

In ASD, a decrease of PV-positive (PV^+^) neurons is seen in post mortem brain tissues of affected individuals, as well as in several ASD mouse models [12–14]. Of importance, a decrease in the number PV^+^ neurons is not necessarily the result of a diminution of PVALB neurons, but may equally result from PV downregulation. In line, PV deficiency in genetically modified mice characterized by unchanged numbers of PVALB neurons [12] (PV+/− and PV−/− juvenile mice) is sufficient to elicit ASD-like behavior [15]. However, the exact pathological mechanisms leading to the clinical symptoms, as well as the specific role of PVALB neurons in the development of ASD, remain to be elucidated. Given the importance of the PVALB neuronal network in regulating cerebral neuronal activity and based on evidence that PVALB neurons are highly affected/impaired in ASD, here we explore the use of induced pluripotent stem cells (iPSC) as means to investigate the role of PVALB neurons in normal neurodevelopment and ASD.

## iPSC: origin and applications

The limited regenerative capacity of neurons strongly affects functional recovery after a cerebral insult (i.e., trauma, stroke, hypoxia). This makes brain repair extremely challenging, as well as the development of therapeutic strategies to treat neurological and also neurodevelopmental diseases. For this aim, cell-based therapies have been developed and represent promising strategies for brain repair. The first approach of a cell-based therapy was initially tested in animal models of Parkinson’s disease (PD) in the 1990s, subsequently with the transplantation of fetal dopaminergic neurons in the striatum of PD patients [16–18]. Despite the improvement of patients after transplantation, the ethical issues and technical challenges associated with fetal transplantation [19] pushed researchers to find more accessible alternatives. In 1998, the derivation of the first human embryonic stem cells (hESC) [20] and their potential to differentiate these cells towards specialized cell types started a revolution in the field of regenerative medicine and brain repair. While the use of hESC in clinical applications has recently increased [21], in 2006, the reprogramming of human skin fibroblasts with four factors inducing pluripotency, i.e., Oct3/4, Sox2, Klf4, and c-MYC [22], initiated the use of induced pluripotent stem cells (iPSC); because of their somatic cell’s origin, the application of iPSC circumvents the ethical concerns pertaining to the use of hESC [23] and are considered as an alternative and dynamic system to investigate cellular, molecular, and functional aspects underlying neurodegenerative and neurodevelopmental diseases.

The potentially high preclinical/clinical relevance of iPSC in the study of cerebral functions and the hope to eventually translate this knowledge into the development of future treatment of clinical conditions [24, 25], necessitates the development of protocols that enable to differentiate iPSC into both pyramidal (excitatory) and inhibitory neurons, closely resembling human cortical networks [26–28]. One of the final goals of neuroscientists working with iPSC is to use these cells not only for the putative treatment of neurodegenerative disorders such as Parkinson’s or Alzheimer’s disease but also as a source of cells that may potentially rescue physiological impairments characterizing neurodevelopmental disorders (e.g., ASD, schizophrenia). Additionally, basic research with such iPSC in vitro models is assumed to lead to mechanistic insights on the functioning of neuronal networks and the interplay between excitatory and inhibitory neurons.

## ASD, iPSC, and parvalbumin interneurons

Although many different hypotheses on the etiology of neurodevelopmental disorders have been put forward—including changes in synapse structure/function, brain connectivity, Ca^2+^ signaling, oxidative stress, neurotrophic factors—perturbations in the so-called excitation/inhibition (E/I) balance are viewed as an essential cause for such disorders [29, 30]. While in earlier times, such an E/I imbalance in ASD was attributed to an increased E/I ratio (i.e., an increase in glutamatergic signaling and/or a decrease in GABAergic signaling) [29], a large body of evidence points towards a crucial role of inhibition in the (homeostatic) maintenance of a constant E/I ratio [31–33]. In cortical networks, inhibition is mediated by GABAergic interneurons. Highly diverse interneuron subpopulations can be distinguished based on their morphological and electrophysiological features, as well as on their connectivity [34, 35]. Also, the expression of specific markers allows for a classification of interneurons in three main subgroups: (1) interneurons expressing the neuropeptide somatostatin (SST), (2) interneurons expressing the ionotropic serotonin receptor 5HT3a (5HT3a), and (3) interneurons expressing the Ca^2+^-binding protein parvalbumin (PV; *PVALB*). PV-expressing interneurons (PVALB neurons) account for ~ 40–50% of all GABAergic interneurons [36] and are essential in maintaining the integrity of neuronal circuitry; their particular fast-firing properties synchronize the electrical activity of cortical networks [8, 10, 37]. Moreover, studies in rodents show that PV modulates short-term synaptic plasticity in cortical, striatal, and cerebellar Pvalb neurons and affects excitability and regularity of firing of striatal Pvalb neurons (for details, see [38]. Interestingly, PVALB neurons seem to be affected primarily in several neurodevelopmental disorders [39]: a decreased number of PV^+^ neurons have been reported in ASD [14] and schizophrenia [40]. Although it was initially assumed that the decrease in PV^+^ neurons was the result of neuronal loss, more recent works indicate that *PVALB* mRNA [41, 42] and PV downregulation [12, 13, 38] are the most likely cause for the observed decrease in the number of PV^+^ neurons.

Given the involvement of interneurons in the abovementioned neuropsychiatric diseases, several efforts have been made to generate PVALB neurons from iPSC [43–45]. Nevertheless, their generation is challenging [46]; although protocols aimed at enriching interneuron subpopulations have been designed [47], the interneurons’ heterogeneity makes the identification of each subtype most demanding.

The origin of PVALB neurons in vivo appears to be the medial ganglionic eminence (MGE), a brain region located in the ventral forebrain during embryonic development [48, 49]. Interneuron progenitors from the MGE tangentially migrate to the neocortex, under the influence of several transcription factors, including DLX1, DLX2, DLX5, NKX2.1, and LHX6, the latter being induced by the morphogen sonic hedgehog (SHH) and present in progenitor cells giving rise to PVALB neurons [48–50]. Hence, overexpression of these transcription factors has been used to differentiate iPSC into cortical interneurons [47, 51, 52]. Although interneuron differentiation is achieved using these protocols, the functional maturation of PVALB neurons (in vitro*)* is usually very long (up to 7 months in certain cases) [47, 51] and the percentage of PVALB neurons within those differentiated iPSC cultures is very low, even after weeks of culture [52]. Recently, Yuan and colleagues established a new protocol that significantly increases the number of PVALB neurons in a shorter time (∼ 80 days) through the induction of the LHX6 transcription factor [45].

Nevertheless, the (still) time-consuming differentiation process of PVALB neurons seriously limits the potential use of iPSC in studying the function of PV and PVALB neurons in neurodevelopmental disorders. To the best of our knowledge, the unambiguous identification of PVALB neurons (among all other cells) in iPSC can be achieved only in differentiated cells and mostly relies on the use of immunohistochemistry; thus, at least part of the differentiated iPSC samples needs to be “sacrificed” for fixation and PV immunostaining. In addition to that, a certain threshold of PV expression has to be reached for the antibody to reliably detect PV. In addition, the fact that monitoring PVALB neurons before complete differentiation is currently impossible obstructs the investigation of the molecular changes that are brought about in these neurons by the onset of PV expression. Given the importance of PVALB neurons in regulating cerebral neuronal activity and their altered functioning in neuropsychiatric diseases [39], we foresee that the development of a methodology allowing for their identification in an unperturbed situation and in real-time in iPSC cultures in vitro would represent a big advantage.

### Generation of tagged-parvalbumin neurons by a CRISPR/Cas9 approach as a tool to follow PVALB neuron cell fate

The CRISPR/Cas9 genome-editing technology (reviewed in [53]) has been broadly used to modify iPSC and recently, many studies successfully demonstrated the generation of human iPSC lines expressing a fluorescent protein that faithfully recapitulates the endogenous expression of a given protein [54, 55]. Through a CRISPR/Cas9 approach, one could insert a reporter cassette (i.e., eGFP, tdTomato), whose expression is driven by the endogenous *PVALB* promoter, allowing to follow the fate of these neurons once PV expression starts. However, it is important to point out that the integration of the eGFP reporter in the *PVALB* locus (similar to the integration of Cre recombinase in the PV-Cre line [56] may modify the transcription/translation of the endogenous *PVALB* gene; therefore, it is important to perform initial experiments to carefully confirm the fidelity of the reporter, both by immunofluorescence and analysis of electrophysiological properties of the labeled presumably PVALB neurons. Although this approach ensures the possibility to monitor and isolate PVALB neurons at an early phase of their maturation, it does not allow to observing the fate of these neurons *before* the onset of PV expression, since the *PVALB* promoter is not active in undifferentiated iPSC. To overcome this limitation and to follow the early steps of the development of PVALB neurons, one could imagine a line, where the PVALB neuron lineage is observable by the expression of a reporter (e.g., GFP, tdTomato) driven by a promoter active in MGE-derived cells (and specific for the PVALB neuron lineage [39]) during differentiation.

We are confident that the development of new tools to indirectly tag PV expression will facilitate the investigation of the role of PVALB neurons and the function of PV itself both in normal neurodevelopment and in neurodevelopmental disorders.

More precisely, the fluorescent tag restricted to PVALB interneurons would allow to specifically access this class of interneurons and profile their development from a molecular point of view, starting at an earlier developmental time point before neuronal maturation (i.e., gene expression, RNA sequencing).

In the long term, comparative analysis of tagged-PV neurons derived from healthy and ASD-patients might eventually reveal novel targets for pharmacological modulation and/or for the development of a platform to test medication/gene interaction for personalized treatment of patients.

## Conclusion

While in disorders such as ASD, schizophrenia, and possibly other neurodevelopmental disorders (e.g., attention deficit hyperactivity disorder, where GABAergic interneurons were found also to play a role [57], the importance of PVALB neurons for proper brain function is undisputed, much more needs to be learned about when and how impairment of these neurons’ structure/function is occurring, subsequently leading to the phenotypic changes observed in vivo in animal models, as well as in affected patients.

The use of the CRISPR/Cas9 technology combined with iPSC to tag PVALB neurons will enable investigations on (1) the molecular and cellular mechanisms that are required for establishing/maintaining a functional PVALB neuronal network in iPSC from healthy donors and (2) will eventually allow to follow the fate of PVALB neurons using ASD patients-derived iPSC cultures. The comparison between “healthy” and “ASD” iPSC would shed light on how a certain mutation in an ASD risk gene affects the development and possibly the function of PVALB neurons, assumingly unraveling the role of these neurons in neurodevelopmental diseases.

## Data Availability

Not applicable

## Abbreviations

ASD: Autism spectrum disorder
E/I balance: Excitation/inhibition balance
iPSC: Induced pluripotent stem cells
MGE: Medial ganglionic eminence
PD: Parkinson’s disease
PV: Parvalbumin
PVALB: Parvalbumin-expressing neurons
